# Different electrostatic forces drive the binding kinetics of SARS-CoV, SARS-CoV-2 and MERS-CoV Envelope proteins with the PDZ2 domain of ZO1

**DOI:** 10.1038/s41598-023-35079-7

**Published:** 2023-05-16

**Authors:** Valeria Pennacchietti, Angelo Toto

**Affiliations:** Dipartimento di Scienze Biochimiche “A. Rossi Fanelli”, Laboratory Affiliated to Istituto Pasteur Italia-Fondazione Cenci Bolognetti, Sapienza Università di Roma, P.le Aldo Moro 5, 00185 Rome, Italy

**Keywords:** Biochemistry, Peptides, Proteins

## Abstract

The Envelope protein (E) is a structural protein encoded by the genome of SARS-CoV, SARS-CoV-2 and MERS-CoV Coronaviruses. It is poorly present in the virus but highly expressed in the host cell, with prominent role in virus assembly and virulence. The E protein possesses a PDZ-binding motif (PBM) at its C terminus that allows it to interact with host PDZ domain containing proteins. ZO1 is a key protein in assembling the cytoplasmic plaque of epithelial and endothelial Tight Junctions (TJs) as well as in determining cell differentiation, proliferation and polarity. The PDZ2 domain of ZO1 is known to interact with the Coronaviruses Envelope proteins, however the molecular details of such interaction have not been established. In this paper we directly measured, through Fluorescence Resonance Energy Transfer and Stopped-Flow methodology, the binding kinetics of the PDZ2 domain of ZO1 with peptides mimicking the C-terminal portion of the Envelope protein from SARS-CoV, SARS-CoV-2 and MERS-CoV in different ionic strength conditions. Interestingly, the peptide mimicking the E protein from MERS-CoV display much higher microscopic association rate constant with PDZ2 compared to SARS-CoV and SARS-CoV-2 suggesting a stronger contribution of electrostatic forces in the early events of binding. A comparison of thermodynamic and kinetic data obtained at increasing ionic strengths put in evidence different contribution of electrostatics in the recognition and complex formation events for the three peptides. Our data are discussed under the light of available structural data of PDZ2 domain of ZO1 and of previous works about these protein systems.

## Introduction

It is a common strategy among pathogens, such as viruses and bacteria, to evolve to display in their proteins consensus sequences that allows them to interact with host proteins^1^, mimicking Short Linear Motifs (SLiMs) that can be recognized and bound by specific protein–protein interactions modules^2^. These interactions, which are at the basis of the regulation of almost all physiological and molecular pathways in the cell, can be disrupted by pathogens eventually leading to the development of diseases^1^.

Coronaviruses (CoV) are enveloped viruses that can infect humans at level of the respiratory system, ranging from infections of the upper respiratory tract, resembling the common cold, to the lower respiratory tract causing bronchitis and pneumonia. The single stranded positive sense RNA genome of about 30 kb of Coronaviruses encodes for a series of non-structural proteins and four major structural proteins, namely the Nucleocapsid protein (N), the Membrane protein (M), the Spike protein (S) and the Envelope protein (E). The E protein is the shortest and most enigmatic of the four structural proteins. It is an integral membrane protein in which three domains can be identified: a hydrophobic transmembrane domain (TMD), responsible of the formation of an alpha-helical structure that undergoes oligomerization and subsequent constitution of an ion channel in the membrane, flanked by a short hydrophilic N-terminal domain (NTD) and the largest hydrophilic C-terminal domain (CTD)^3^.

The CTD of E proteins of SARS-CoV, SARS-CoV-2 and MERS-CoV (three members of the Coronaviruses family) has been established to display, at its C terminus, a PDZ-binding motif (PBM), that is a consensus sequence recognized and bound by PDZ domains^4,5^. PDZ domains are the most abundant protein–protein interaction modules in the human proteome, representing a common target of viral pathogens, from adenoviruses, rabies, HPV to influenza^6,7^. The ability of E protein to interact with PDZ domain containing proteins is well established and it has been reported as a key event in the virulence mechanisms of the viruses, as well as in virion trafficking, assembling and budding^8^. Interestingly, engineered viruses lacking the C-terminal PBM in the E protein resulted to be less virulent in in vivo experiments, with the tendency to acquire alternative PBMs after several cell passages, confirming the importance of PDZ-mediated interactions^9,10^.

One of the cellular targets of the PBM of the E protein is ZO1^11^. ZO1 is a PDZ containing protein with a critical role in assembling the cytoplasmic plaque of epithelial and endothelial Tight Junctions (TJs) as well as in determining cell differentiation, proliferation and polarity^12,13^. ZO1 exerts its scaffolding functions through its three PDZ domains and the SH3 domain, that mediate protein–protein interactions and govern the spatial arrangement of the cytosolic protein complex of TJs^12,14–16^. Interestingly, the interaction between the PDZ2 domain of ZO1 and the E protein has been suggested to be fundamental in disrupting the delicate and sophisticated mechanism of TJs assembling, possibly determining the characteristic epithelial lung damages and pulmonary disfunction that may occur upon and after CoVs infections^17–20^.

Although the interaction occurring between E protein and the PDZ2 of ZO1 is known, the molecular details of such interactions are not completely understood. In this paper we investigate the binding reaction occurring between peptides mimicking the C-terminal region of the Envelope proteins from SARS-CoV (sequence NSSEGVPDLLV), SARS-CoV-2 (sequence NSSRVPDLLV) and MERS-CoV (sequence SKPPLPPDEWV) and the PDZ2 domain of ZO1 from kinetic and thermodynamic perspectives. The analysis of stopped-flow binding kinetic data demonstrates the ability of PDZ2 to bind the Envelope proteins from MERS-CoV with much higher kinetic parameters (microscopic association and dissociation rate constants) compared to SARS-CoV and SARS-CoV-2. Moreover, a comparison of thermodynamic equilibrium and kinetic data obtained at different ionic strength conditions is provided, highlighting dissimilar contribution of electrostatic charges in the early recognition event and in the late complex stabilization event for the three peptides. Data are discussed under the light of available structural data of PDZ2 domain of ZO1 and of previous works about these protein systems.

## Results and discussion

### Binding kinetics between PDZ2 and E peptides

To spectroscopically monitor the kinetics of the binding between PDZ2 of ZO1 and peptides mimicking Envelope protein we resorted to measure them through Fluorescence Resonance Energy Transfer (FRET), following an approach successfully used in the past^19^. We engineered a pseudo wild-type variant of PDZ2, by mutating the F residue in position 207–W (F207W) to act as a donor group, the acceptor being a dansyl group covalently linked to the N terminus of the peptides. Kinetic binding experiments were performed with a SX-18 Stopped-Flow apparatus (Applied Photophysics) by rapidly mixing a constant concentration of peptide mimicking the E proteins versus increasing concentrations of PDZ2 Y207W. The buffer used was Hepes 50 mM pH 7.0, and the temperature was set to 10 °C. Samples were excited at 280 nm and fluorescence emission was collected by using a 475 nm cut-off filter. For each experiment performed, 3–5 individual traces were acquired and then averaged. An increase in FRET signal could be monitored upon binding. All the averages collected were satisfactorily fitted with Eq. (1)

1 $$y= A exp (-{k}_{obs}\cdot t)+cost$$

to calculate the observed rate constant of the reaction (*k*_obs_).

**Figure 1:**
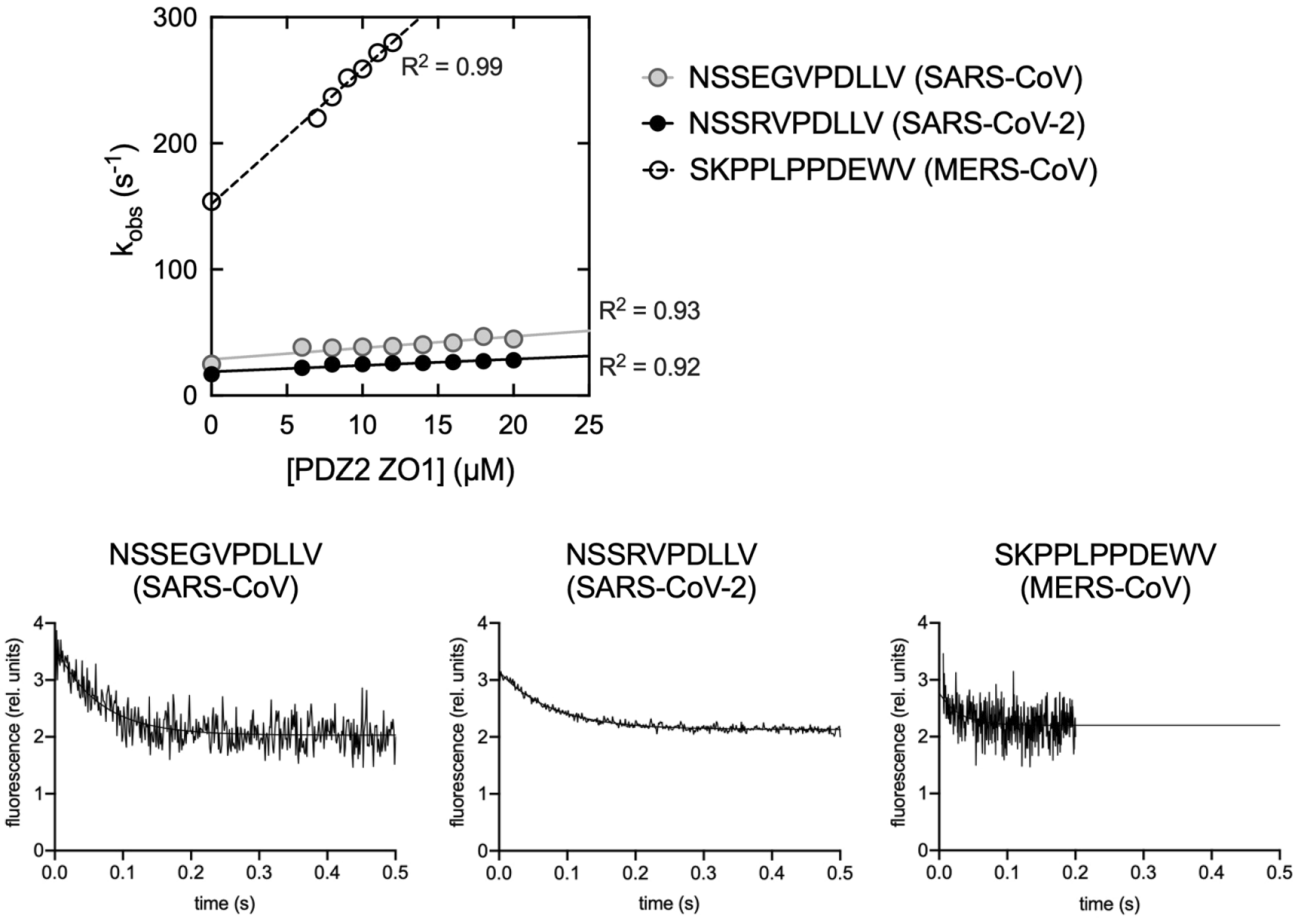
Top—kinetics of the binding reaction between peptides mimicking the C-terminal region of the Envelope proteins from SARS-CoV, SARS-CoV-2 and MERS-CoV versus different concentrations of PDZ2 domain of ZO1. Straight lines are the best fit to a linear equation. R^2^ values for linear regression are reported. Bottom—as described in the text, points at 0 concentration of ligand were measured in separated displacement experiments. Lines are the best fit to a single exponential equation.

To obtain quantitative kinetic information about the binding mechanism of PDZ2 with the three different peptides we analyzed the dependences of the calculated *k*_obs_ as a function of the concentration of PDZ2 (reported in Fig. 1). Data were fitted with Eq. (2)

2 $${k}_{obs}={k}_{on}[PDZ2]+{k}_{off}$$

which allows to calculate the microscopic association rate constant (*k*_on_) as the slope of the straight line. Linear analysis of *k*_obs_ under pseudo-first order conditions would allow to extrapolate the microscopic dissociation rate constants (*k*_off_) as the intercept to the y-axis. Although this procedure is correct in theory, the high experimental error that usually arises could jeopardizes a reliable calculation of *k*_off_. Thus, to directly determine *k*_off_ values we resorted to perform displacement experiments, in which a pre-incubated complex of dansylated E peptides (at final concentration of 4 µM) and PDZ2 (at final concentration of 20 µM) were rapidly mixed with different concentrations, in high excess, of non-dansylated E peptides (ranging from 50 to 100 µM). In agreement with theory^22^ the observed rate constants were found insensitive to the displacer concentrations. The displacement traces obtained for the three E peptides are reported in Fig. 1 (bottom panel). The *k*_on_ and *k*_off_ values obtained are reported in Table 1, together with the equilibrium dissociation rate constant K_D_, calculated as *k*_off_/*k*_on_.

**Table 1:** Kinetic and affinity parameters obtained from linear fitting of data reported in Fig. 1 (top panel) and Fig. 2.

[NaCl]	*k*_on_ (µM^−1^ s^−1^)	*k*_off_ (s^−1^)	*K*_D_ (µM)
SARS-CoV E peptide			
0 M	1.9 ± 0.2	9.0 ± 0.1	4.7 ± 0.2
0.030 M	1.4 ± 0.2	7.0 ± 0.1	5.0 ± 0.2
0.075 M	0.8 ± 0.2	6.9 ± 0.1	8.6 ± 0.3
0.150 M	0.6 ± 0.1	6.2 ± 0.1	10.3 ± 0.2
SARS-CoV-2 E peptide			
0 M	0.6 ± 0.1	9.1 ± 0.2	15.2 ± 0.3
0.030 M	0.6 ± 0.1	9.5 ± 0.1	15.8 ± 0.2
0.075 M	0.5 ± 0.1	8.7 ± 0.2	17.4 ± 0.1
0.150 M	0.4 ± 0.1	6.8 ± 0.1	17.0 ± 0.2
MERS-CoV E peptide			
0 M	10.7 ± 0.3	154 ± 3	14 ± 1
0.030 M	*	*	19 ± 3
0.075 M	*	*	34 ± 4
0.150 M	*	*	29 ± 3

Equilibrium dissociation rate constants K_D_ were calculated as *k*_off_/*k*_on_ or from equilibrium binding experiments.

Data reported in Fig. 1 and Table 1 highlight the ability of the Envelope protein from MERS-CoV to reach a more rapid equilibrium with the PDZ2 of ZO1 compared to the SARS-CoV and SARS-CoV-2 Envelope proteins, and a higher affinity for PDZ2 by a factor of ~ 2. It is worth noticing, however, that the measured affinities (K_D_) are all in the µM range. These values are in agreement to what is typically observed in SLiMs interactions, usually characterized by a medium–low affinity in the range of 1–500 µM^23^.

The analysis of kinetic data put in evidence that, while SARS-CoV and SARS-CoV-2 E peptides show very similar binding kinetics, MERS-CoV E peptide reports a dramatically higher microscopic association and dissociation rate constants. In particular, the *k*_on_ measured for MERS-CoV E peptide (10.7 ± 0.3 µM^−1^ s^−1^) is one order of magnitude higher compared to the *k*_on_ of SARS-CoV (1.9 ± 0.2 µM^−1^ s^−1^) and two orders of magnitude higher compared to the *k*_on_ of SARS-CoV-2 (0.6 ± 0.1 µM^−1^ s^−1^). These results may be explained by the formation of more favorable electrostatic attraction occurring in MERS-CoV E peptide than what happens for SARS-CoV and SARS-CoV-2^24,25^. PDZ domains are known to recognize mainly the C-terminal carboxylate group by a “carboxylate-binding loop” conserved in the PDZ domain family^26^, with the last five residues, conventionally numbered from 0, the C-terminal, to − 4, tuning their binding specificity and affinity. Typically, position 0 and − 2 are the most important in that sense, with a hydrophobic residue at position 0 required to fit into a hydrophobic pocket of PDZ domains, and a polar residue often found at position − 2 of the ligand^27^. The presence of a glutamate residue in position − 2 in the MERS-CoV E protein may be causative of the faster association reported in Fig. 1 compared to SARS-CoV and SARS-CoV-2 E proteins, that present a hydrophobic leucine in the same positions.

### Ionic strength dependence of the binding reaction

**Figure 2:**
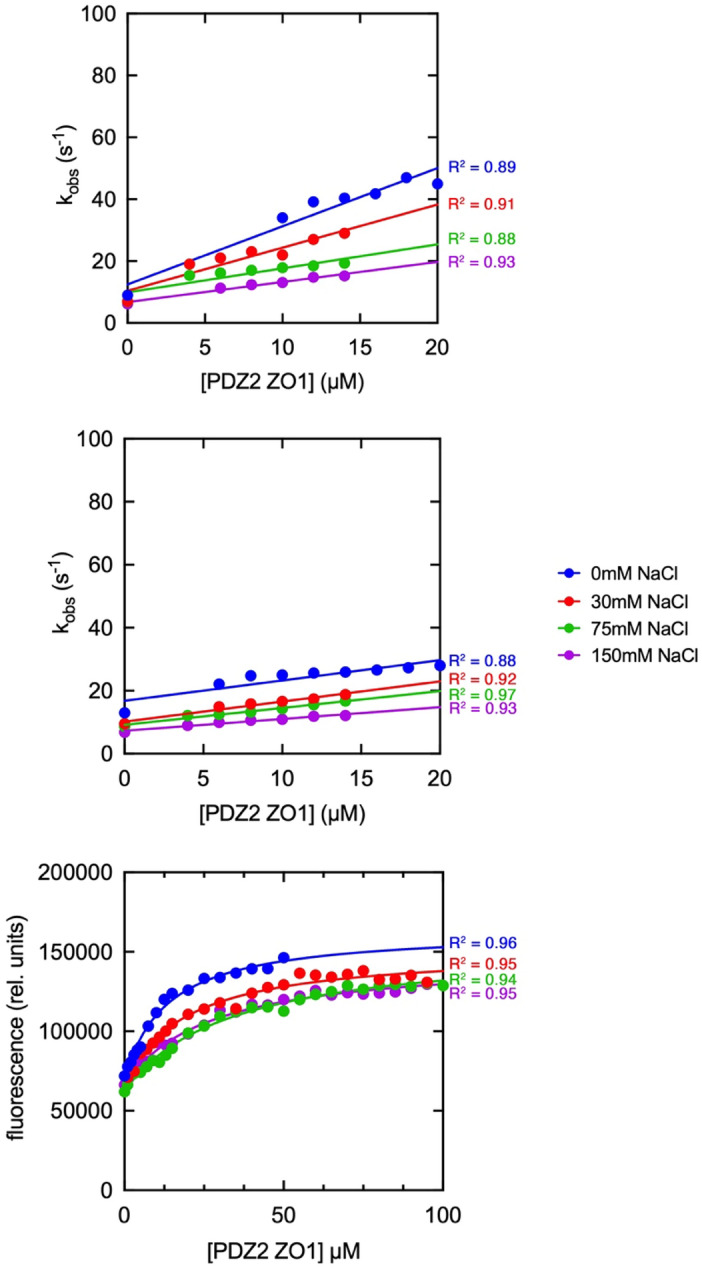
Ionic strength dependence of the binding reaction between SARS-CoV, SARS-CoV-2 and MERS-CoV E peptides versus different concentrations of PDZ2 domain of ZO1. Top and center panel represent stopped-flow kinetic experiments, while bottom panel represents equilibrium binding experiments at different concentrations of NaCl added to the experimental buffer. Lines are the best fit to a linear (left and center panels) and hyperbolic (right panel) equations. R^2^ values are reported for all the fitting processes.

To further investigate the mechanism of interaction between PDZ2 and the E peptides we resorted to monitor the binding kinetics at different experimental conditions, changing the ionic strength of the solution. Experiments were performed in buffer Hepes 50 mM pH 7.0 and progressively adding increasing concentration of NaCl (ranging from 30 to 150 mM). While for the peptide mimicking MERS-CoV E protein the addition of NaCl to the buffer caused a dramatic decrease of the amplitude of kinetic traces, such that we could not obtain any reliable kinetic data, the observed rate constants for the binding of SARS-CoV and SARS-CoV-2 E peptides at different NaCl concentrations could be measured (Fig. 2). Points at 0 µM [PDZ2] correspond to the *k*_off_ and were obtained by displacement experiments and data were satisfactorily fitted with Eq. (2). To measure the effect of NaCl concentration on the affinity of PDZ2 for MERS-CoV E peptide we performed equilibrium binding experiments by keeping the E peptide at constant concentration (1 µM) and following the change of FRET signal at increasing [PDZ2]. Data obtained at 500 nm wavelength were then plotted and fitted with a hyperbolic function which returned the binding K_D_.

**Figure 3:**
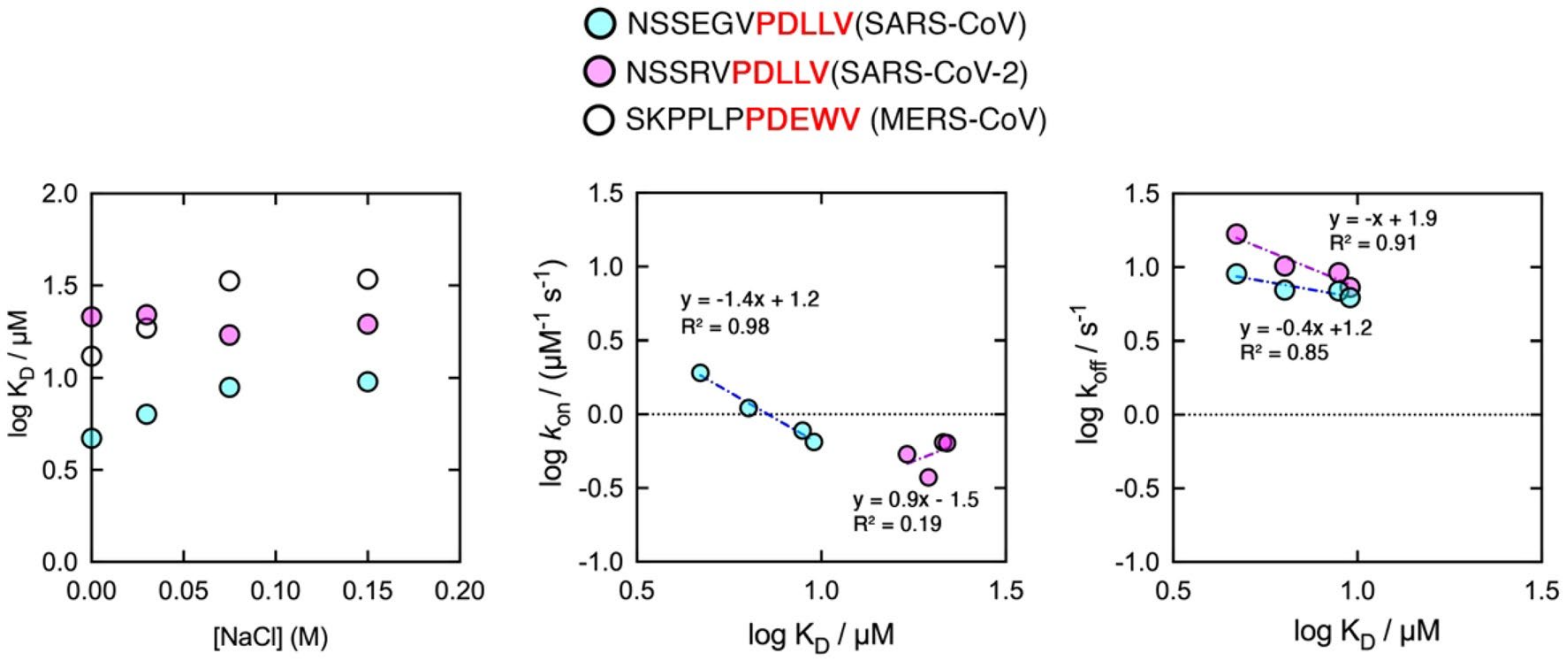
Left panel—dependence of equilibrium dissociation rate constant (*K*_D_) as function of NaCl concentration added to the experimental buffer. Data show that the stability of the complex formed by SARS-CoV-2 E peptide appears to be mostly insensitive to increasing ionic strengths, while an increasing K_D_ is appreciable for SARS-CoV and MERS-CoV peptides at increasing [NaCl]. Center and right panel—dependence of microscopic association (*k*_on_) and dissociation (*k*_off_) rate constants as function of K_D_. Lines represent the best fit to a linear equation. R^2^ values are reported. For both SARS-CoV and SARS-CoV-2 E peptides *k*_*o*ff_ dependences are well described by a linear regression. On the other hand, only the *k*_*on*_ dependence of SARS-CoV peptide is well fitted by a linear equation.

The dependence of the logarithm of calculated K_D_ as function of the concentration of NaCl concentration added to the buffer are shown in Fig. 3, and their values are reported in Table 1. Increasing the ionic strength of the solution decreased the affinity of PDZ2 for the SARS-CoV and MERS-CoV E peptides, while the affinity for SARS-CoV-2 E peptide appears mostly unaffected. Moreover, a deeper analysis of kinetic data highlights that the decrease in affinity for SARS-CoV E peptide is due to a stronger effect on *k*_on_. It is of interest to notice that the dependence of the log *k*_on_ as function of log K_D_ for SARS-CoV E peptide can be satisfactorily fitted with a linear equation that reported a R^2^ = 0.98, while such correlation is absent in the case of SARS-CoV-2 peptide (R^2^ = 0.19). These data must be interpreted comparing the sequences of SARS-CoV and SARS-CoV-2 E peptides which possess identical PDZ binding motifs (highlighted in red in Fig. 3). Thus, under the light of PDZ binding properties that have been previously discussed (and reviewed here^27^), the early events of the binding of the C-terminal portion of the E protein by the PDZ2 domain of ZO1 may be regulated by transient electrostatic interactions occurring outside of the canonical PDZ binding pocket, which then stabilizes the final docking of the protein complex. Notably, a computational analysis of the binding mechanism of the PDZ domain from protein tyrosine phosphatase 1E (PTP1E) highlighted the formation of non-native electrostatic interactions in the early events of binding that were not present in the final bound complex^28^. Our results let us hypothesize that the formation of non-native contacts in the encounter complex might be a shared characteristic of different PDZ domains.

**Figure 4:**
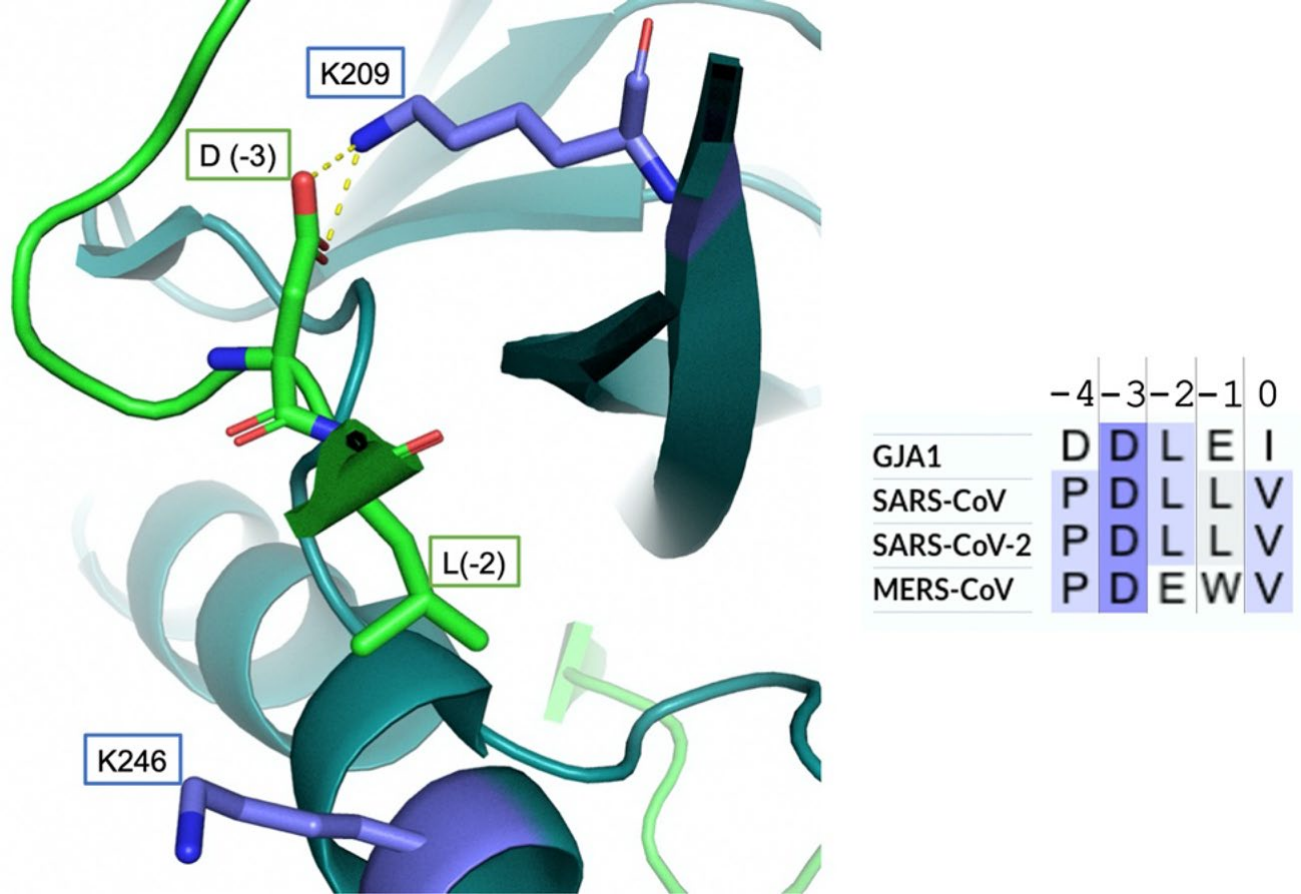
Structural analysis of the binding complex between the PDZ2 domain of ZO1 and a peptide mimicking the physiological ligand Connexin-43 (Cx43-GJA1) (PDB: 3CYY) and sequence alignment with SARS-CoV, SARS-CoV-2 and MERS-CoV E proteins PBMs. An electrostatic interaction between the − 3 position of the ligand and the K209 residue is highlighted as yellow dashed lines. The − 2 position of the ligand and K246 residue of PDZ2 are also highlighted. The presence of a glutamate residue in the − 2 position of the MERS-CoV E peptide may be causative of the formation of a second salt bridge, which may explain the faster association kinetics observed in the binding experiments.

Structural information about the PDZ2 domain of ZO1 in complex with a peptide mimicking the physiological ligand Connexin-43 (Cx43–GJA1) are available (PDB: 3CYY). An analysis of the structure of the PDZ2:Cx43 complex highlights the formation of a salt bridge between the residue K209 of the PDZ domain and the D in position − 3 of the ligand (Fig. 4). Interestingly, this residue appears to be conserved in all the three PBMs of the Envelope proteins from SARS-CoV, SARS-CoV-2 and MERS-CoV, suggesting an evolutive pressure on the gene encoding for the Envelope protein of Coronaviruses to display a SLiM able to bind the PDZ2 domain of ZO1 and hijack its physiological functions.

Another interesting point is that Cx43 possesses a hydrophobic residue (L) at − 2 position, analogously to SARS-CoV and SARS-CoV-2 E proteins. This leucine is at the interface with a positively charged residue (K246) of PDZ2 that might generate a second salt bridge with the E residue at position − 2 of MERS-CoV E protein, possibly explaining the faster association observed in kinetic binding experiments. Moreover, K246 appears to form an intramolecular salt bridge with E250 residue that may be influenced by the presence of NaCl. However, increasing the ionic strength did not affect the thermodynamic stability of PDZ2 domain (see Supplementary Materials).

Regarding the functional aspects of the formation of a second salt bridge, we can only speculate. As we mentioned in the Introduction, a common strategy for pathogens is to develop the ability to bind intracellular proteins to hijack physiological and molecular pathways in the cell. So that, when a protein can interact with more than one partner with similar affinities, protein concentrations and kinetics are fundamental in determining complex formations. Regarding the E protein, its interactions with PDZ containing proteins involved in Tight Junctions (TJs) formation is well documented^11,18–20^. Our laboratory recently published an ultrafast kinetic analysis of the same peptides used in this work (mimicking SARS-CoV and SARS-CoV-2). Data reported much higher kinetic parameters in the binding with the PDZ domain of PALS1^19^ (another protein with an important role in the formation of cellular tight junction) but with lower affinities, compared to the PDZ2 domain of ZO1. While the role of PALS1 and ZO1 in TJs formation is established, their exact molecular function is not completely understood. PDZ domains and SH3 domain of ZO1 allows it to bind several different partners and mediate the formation of TJs supramolecular complexes^29^, however knock-out mice of ZO1 can form perfectly functional TJs, although with a delay in junction assembly^30^. In the case of MERS-CoV Envelope protein, our data show that the complex formation is characterized by a rapid equilibrium, that can prevent ZO1 to interact with its physiological partners possibly provoking a more effective disruption of the interactions in which ZO1 is involved, without completely compromising the TJs formation. Extensive site-directed mutagenesis, as well as a structural characterization of the PDZ2 domain in complex with Coronavirus Envelope proteins are strongly demanded in order to confirm our hypothesis, pinpoint PDZ residues directly involved in the recognition of viral peptides, and finally to pave the way towards the development of pharmaceutical strategies aimed to block this important interaction for the replication of Coronaviruses.

## Conclusions

The ability of Coronaviruses Envelope protein to interact with different PDZ domain containing proteins^17–19^ is a known key feature for viruses development in the intracellular environment. In this work, we show that electrostatic forces drive the binding of the Envelope proteins from SARS-CoV, SARS-CoV-2 and MERS-CoV with the second PDZ domain of ZO1. Although there are no structural data about these protein complexes, by analyzing the structure of PDZ2 domain with a physiological ligand and by comparing the sequences of the C-terminal portion of the three Envelope proteins, we propose an explanation for the highly different binding kinetics observed. In particular, the formation of salt bridges appears to be at the basis of faster association observed for MERS-CoV E peptide compared to SARS-CoV and SARS-CoV-2. Interestingly, the different effect of salt concentration on the binding of SARS-CoV and SARS-CoV-2 E peptides suggests the formation of different transient electrostatic interactions that may occur outside of the PDZ domain binding pocket. Altogether, our data and the conclusion gathered represent a step forward in the understanding of the mechanism of interaction of Coronaviruses Envelope protein with the PDZ2 domain of ZO1 and for future structural analysis aimed to characterize such complexes.

## Materials and methods

### Protein expression and purification

The construct encoding the PDZ2 domain of ZO1 protein was subcloned in a pET28b+ plasmid vector and then transformed in Escherichia coli cells BL21 (DE3). Bacterial cells were grown in LB medium, containing 30 μg/mL of kanamycin, at 37 °C until OD_600_ = 0.7–0.8, and then protein expression was induced with 0.5 mM IPTG. After induction, cells were grown at 25 °C overnight and then collected by centrifugation. The pellet was resuspended in buffer 50 mM TrisHCl, 300 mM NaCl, Imidazole 10 mM, pH 8.0, adding one antiprotease tablet (Complete EDTA-free, Roche). After sonication and centrifugation, the soluble fraction from bacterial lysate was loaded onto a Ni-charged HisTrap Chelating HP (GE Healthcare) column equilibrated with 50 mM TrisHCl, 300 mM NaCl, Imidazole 10 mM, pH 8.0. Eluition was obtained by using an ÄKTA-prime system, with a gradient of Imidazole from 0 to 1 M. Fractions were analyzed through SDS-PAGE. Fractions containing the protein were collected and the buffer was exchanged to 50 mM Hepes, 150 mM NaCl, pH 7.0 by using a HiTrap Desalting column (GE Healthcare). The purity of the protein was analyzed through SDS-page. Peptides mimicking the C-terminal region of the Envelope protein from SARS-CoV, SARS-CoV-2 and MERS-CoV, with and without the dansyl N-terminal modification, were purchased from GenScript.

### Equilibrium binding experiments

Equilibrium binding experiments were carried out on a Fluoromax single photon counting spectrofluorometer (Jobin-Yvon, NJ, USA), by mixing a constant concentration of dansylated MERS-CoV E peptide with increasing concetrations of PDZ2 F207W. Experiments were performed at 10 °C, using a quartz cuvette with a path length of 1 cm, in 50 mM Hepes pH 7.0 (with the addition of 0.030 M, 0.075 M and 0.15 M NaCl) measuring the change in FRET signal. The excitation wavelength was 280 nm and fluorescence spectra were recorded between 450 and 550 nm.

### Stopped-flow binding and displacement experiments

Kinetic binding experiments were performed on an Applied Photophysics SX-18 stopped-flow apparatus (Applied Photophysics). Pseudo-first order binding experiments were performed mixing a constant concentration (2 μM) of dansyl-E peptide with increasing [PDZ2]. Samples were excited at 280 nm, and the emission fluorescence was recorded by using a 475 nm cutoff filter. Experiments were performed at 10 °C in buffer Hepes 50 mM pH 7.0.

As detailed in the text, microscopic dissociation rate constants were measured by performing displacement experiments. A preincubated complex of PDZ2 domain (at final concentration of 4 µM) and PDZ2 (at final concentration of 20 µM) were rapidly mixed with different concentrations, in high excess, of non-dansylated E peptides (ranging from 50 to 100 µM). Samples were excited at 280 nm and fluorescence emission was collected by using a 475 nm cutoff filter.

## Supplementary Information

Supplementary Information.

## Data Availability

The datasets generated during and/or analyzed during the current study are available from the corresponding author on reasonable request.
