# Supplementary material for: Different electrostatic forces drive the binding kinetics of SARS-CoV, SARS-CoV-2 and MERS-CoV Envelope proteins with the PDZ2 domain of ZO1
Source: Sci Rep. 2023 May 16;13:7906. doi: 10.1038/s41598-023-35079-7 (PMC10186300; doi:10.1038/s41598-023-35079-7)
Supplement: Supplementary file 1 — Supplementary Information. [file 41598_2023_35079_MOESM1_ESM.docx]

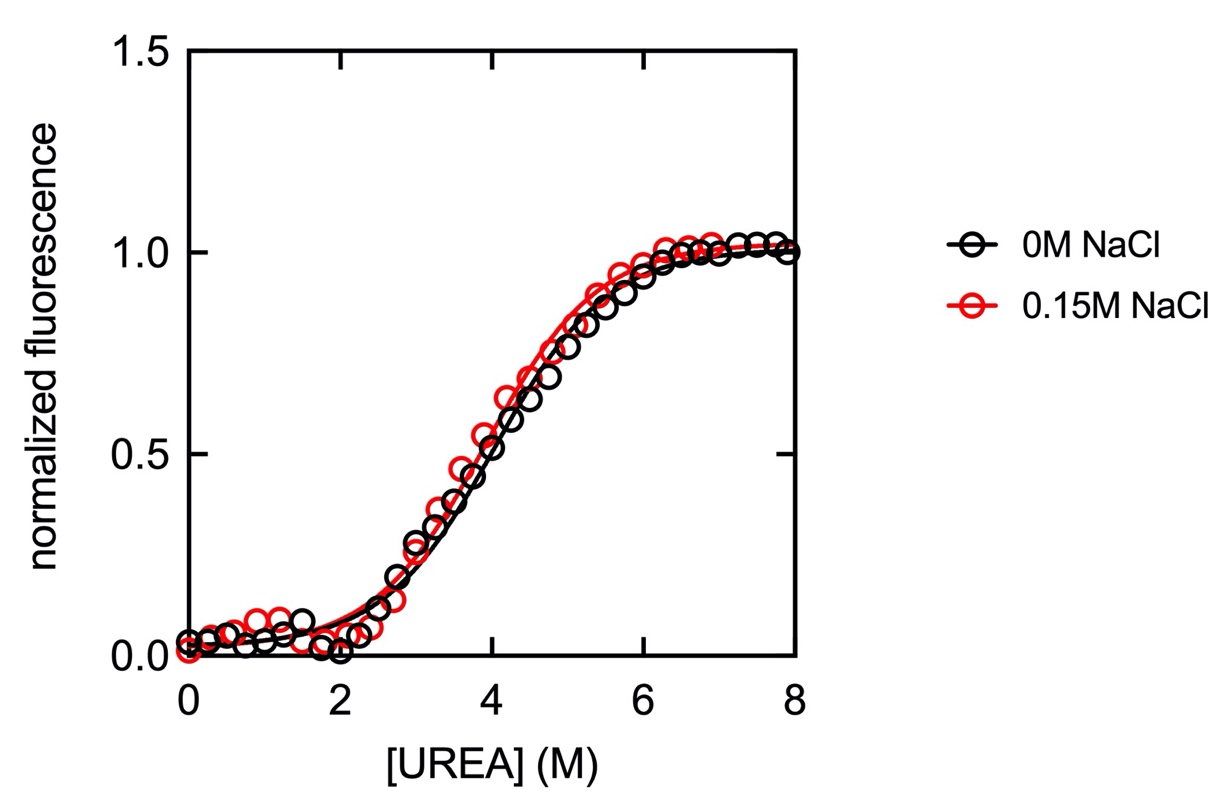


Figure S1 – Equilibrium denaturation of the PDZ2 domain, obtained by challenging a constant concentration of protein with increasing concentration of urea, and following the change in intrinsic fluorescence (normalized fluorescence at 350nm). The experiment was performed in buffer Hepes 50mM pH 7.0, at 10°C, in the absence (black) and presence (red) of 0.15M NaCl. Samples were excited at 280nm, fluorescence was collected between 300nm and 400nm. Data show that the presence of salt does not alter the stability of the domain. Lines represent the best fit to a sigmoidal equation.


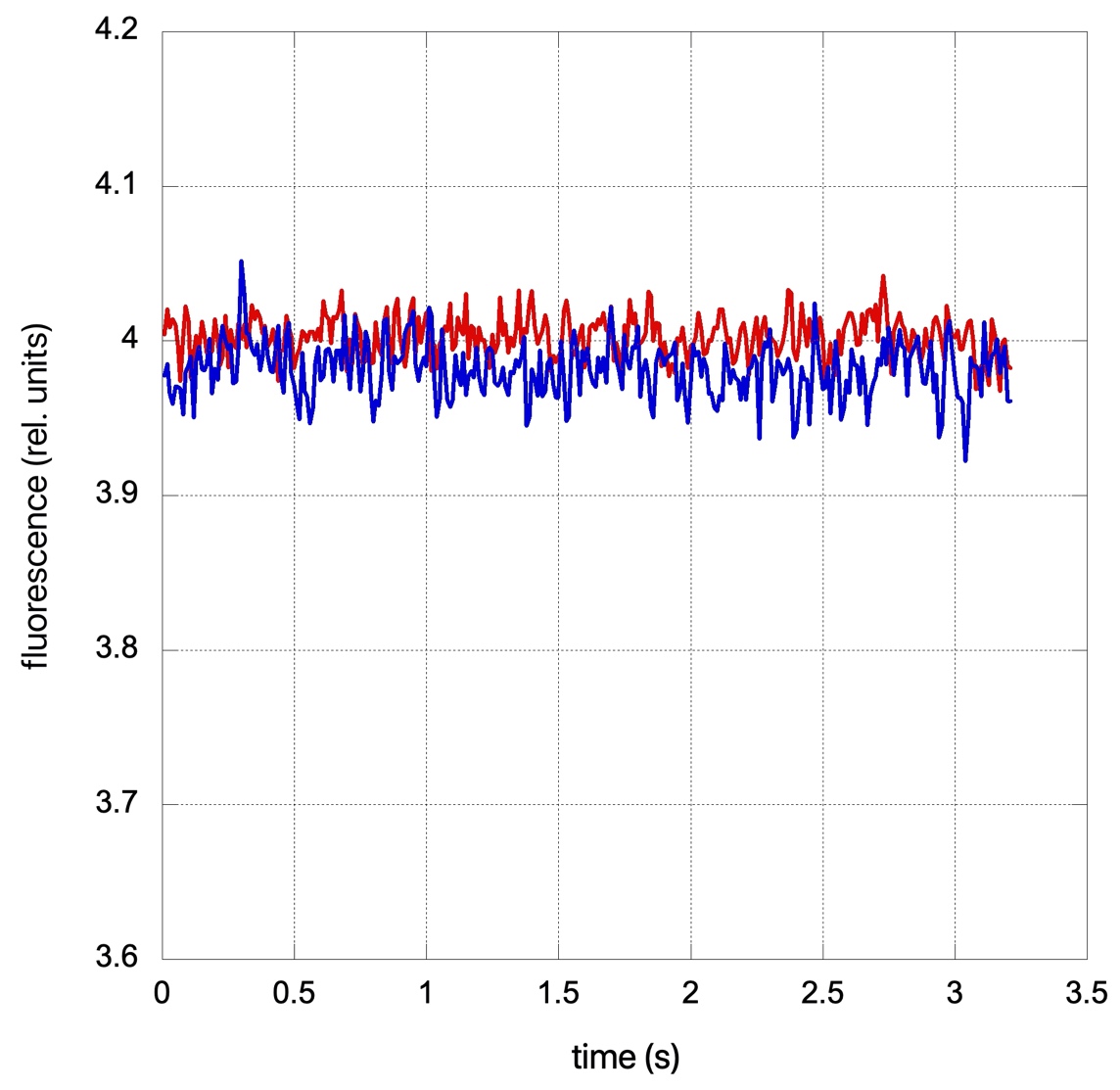


Figure S2 – Binding kinetic experiment performed at the stopped-flow as a negative control, between the pseudo wild-type PDZ2 domain of ZO1 (2µM) and two different concentrations (10µM in red, and 20µM in blue) of a peptide not displaying a PDZ-binding motif (sequence ENVGLMQQQK). Data show that there is no change in fluorescence upon binding. Experimental conditions were the same used in the paper, in the absence of NaCl.
